# Pathophysiology of Trans-Synaptic Adhesion Molecules: Implications for Epilepsy

**DOI:** 10.3389/fcell.2018.00119

**Published:** 2018-09-21

**Authors:** Adam Gorlewicz, Leszek Kaczmarek

**Affiliations:** Laboratory of Neurobiology, Nencki Institute of Experimental Biology, Polish Academy of Sciences, Warsaw, Poland

**Keywords:** synaptic plasticity, autism spectrum disorders, epileptogenesis, neuronal development, neuropsychiatric disorders

## Abstract

Chemical synapses are specialized interfaces between neurons in the brain that transmit and modulate information, thereby integrating cells into multiplicity of interacting neural circuits. Cell adhesion molecules (CAMs) might form trans-synaptic complexes that are crucial for the appropriate identification of synaptic partners and further for the establishment, properties, and dynamics of synapses. When affected, trans-synaptic adhesion mechanisms play a role in synaptopathies in a variety of neuropsychiatric disorders including epilepsy. This review recapitulates current understanding of trans-synaptic interactions in pathophysiology of interneuronal connections. In particular, we discuss here the possible implications of trans-synaptic adhesion dysfunction for epilepsy.

## Introduction

Synaptic connections are important defining features of neurons. They continue to be formed and eliminated throughout the development and postnatal period allowing neurons spreading and processing complex patterns of impulses that underlie cognition. Synapses must maintain and strictly regulate the positioning of two opposite molecular architectures – their receptor and channel specification at the postsynaptic site, and the highly dynamic system of neurotransmitter-containing vesicles at the presynaptic site. Moreover, the alignment of both synaptic constituents must be coordinated in time during junction formation, thus neurotransmitters released from the pre-synaptic membrane could act on receptors located at the post-synaptic site in order to ensure fine flow of information between neurons (Harris and Littleton, 2015).

The biochemical and ultrastructural studies of a synapse suggest that adhesion might be critical for the proper assembly and molecular composition of pre- and postsynaptic specializations (Lučić et al., 2005; Zuber et al., 2005). In fact, electron microscopy revealed that upon biochemical synaptosome (isolated nerve terminal) fractionation, both pre- and postsynaptic constituents remain intact (Evans, 2015). The concept of adhesion-mediated synapse stabilization has been supported by demonstrations that synaptic formation can be facilitated by synaptically localized cell adhesion molecules (CAMs) that cooperate across the synaptic junction (Perez de Arce et al., 2015). Trans-synaptic CAMs govern different aspects of synaptic plasticity. They organize synapse formation, control synapse morphology, and receptor function, as well as are engaged in synaptic elimination (Bhaskar et al., 2010; Siddiqui and Craig, 2011). An aim of this review is to recapitulate current understanding of trans-synaptic interactions in pathophysiology of inter-neuronal connections that underlies epileptogenesis and epilepsy.

The crucial role of trans-synaptic CAMs in shaping synapses is also reflected by the fact that many of these molecules have been linked to synaptopathies in neuropsychiatric disorders, including epilepsy. Epilepsy is a condition that has an impact on more than 60 million people throughout the world (Moshé et al., 2015). The disorder is characterized by the presence of recurrent seizures and such comorbidities as cognitive deficits. The mechanisms that underlie these epileptic abnormalities remain largely unknown (Yuen et al., 2018). Since both seizures and cognition require transfer of information throughout synapses, it is justified to formulate the hypothesis that any dysregulation of trans-synaptic complexes might potentially contribute to development and maintenance of epilepsy and its comorbidities. The experimental evidence to support this notion is growing and gains increasing attention, therefore, another aim of the review is to summarize the studies demonstrating possible role of trans-synaptic adhesion in epilepsy. Importantly, epilepsy and its development named epileptogenesis, provide particularly attractive neuropsychiatric condition to study as they can be effectively investigated using animal models (Lévesque et al., 2016).

At least to some extent, plasticity of interneuronal connections seems to be based on synaptically implemented and strictly controlled enzymatic digestion of trans-synaptic CAMs and their adhesive contacts by extracellular proteases (Ethell and Ethell, 2007; Benson and Huntley, 2012; Shinoe and Goda, 2015; Vafadari et al., 2016). The biological consequences of regulated trans-synaptic CAMs cleavage on physiological and pathological changes remains insufficiently understood. We wish to acknowledge here the role of different extracellular proteases in cleavage of trans-synaptic CAMs and describe their contribution to epileptogenesis and epilepsy.

## Pathophysiology of Trans-Synaptic CAMs

Each chemical synapse is composed of a patch of presynaptic cell membrane from one neuron facing a patch of postsynaptic cell membrane from a second neuron. The space between them is called a synaptic cleft. The presynaptic side of the synapse harbors the molecular machinery essential for a turnover of neurotransmitter-containing vesicles. Its role is to initiate synaptic responses. The postsynaptic side of the synapse contains receptors and ion channels and triggers downstream cellular signals. During synaptic activity presynaptic neuron executes the release of neurotransmitter molecules into the synaptic cleft, then their binding to the postsynaptic receptors activates channels and evokes molecular responses in the postsynaptic neuron. Apart from signal transmission a role of synapses is to wire neurons into specific functional circuits. Synaptic connections are very dynamic and as a result of synaptic activity they undergo structural and functional changes – a process that is called synaptic plasticity. Synaptic plasticity involves alterations of molecular components present at a synapse, which leads to changes in synaptic geometry and communication efficacy. The phenomenon of synaptic plasticity is widely involved in synapse formation, maintenance and elimination and underlies learning, memory and regenerative processes (Harris and Littleton, 2015). Understanding of how a complex molecular signature of an appropriate synaptic connection is established, maintained, and modified over time remains a major research challenge.

The trans-synaptic CAMs play particularly important role in regulation of pre- and postsynaptic molecular assembly during diverse steps of synapse lifespan. During contact establishment at the site of future synapse, the trans-synaptic CAMs mediate recognition of partner cells. Then, they ensure functional specification of developing synaptic contact by the recruitment of molecular components to active zones, and postsynaptic densities, which eventually leads to the different types of functional synapses with distinct physiological properties (Washbourne et al., 2004).

### Dynamic Reorganization of the Synaptic CAMs by Local Proteolysis

The use of trans-synaptic adhesion term here might be, however, misleading because these molecules are not only static structural elements of adhesion, but also dynamic modulators of continual morphological and functional synaptic plasticity (Holtmaat and Svoboda, 2009; Kasai et al., 2010; Chen et al., 2014; Gjørlund et al., 2017). Trans-synaptic CAMs modulate the synapse structure and function with final effect on efficacy of interneuronal connections in line with Konorski/Hebb theorem (Konorski, 1948; Hebb, 1949; Huber and Menzel, 2004). This suggests that trans-synaptic dimers are natural effective means to facilitate molecular changes in synaptic contacts in response to neuronal activity, which might ensure specific retrograde and anterograde modulation of synaptic signal transmission. Indeed, studies on perisynaptic proteolytic cleavage of CAMs provide the promising insights into the possible mechanisms in this regard. Several, possibly complementary, mechanisms have been proposed, that involve regulated proteolysis of trans-synaptic CAMs, including reduced synaptic stability, production of the truncated signaling ectodomains, and increased concentration of transcription-influencing intracellular fragments (Nagappan-Chettiar et al., 2017).

**FIGURE 1:**
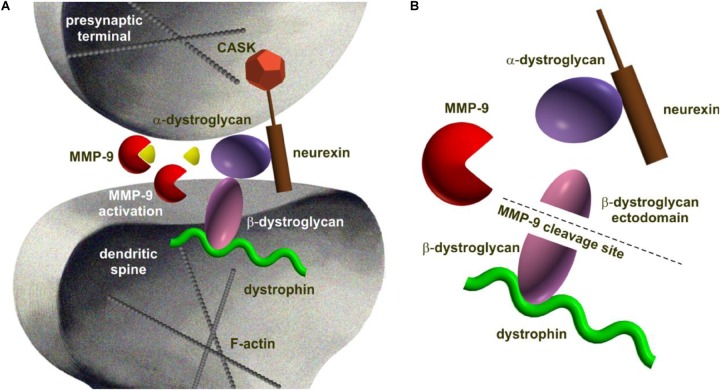
Schematic representation of dystroglycan complex being processed by MMP-9 at the synapse as an example of proteolytic cleavage of trans-synaptic interaction. **(A)** Dystroglycan is an ubiquitous membrane protein associated to dystrophin. It is composed of α- and β-subunits. The α-dystroglycan is a highly glycosylated extracellular component, whereas transmembrane β-dystroglycan forms a bridge between α-subunit and the cytoskeleton. Like neuroligin, dystroglycan binds specifically to neurexins (Sugita et al., 2001). **(B)** MMP-9-mediated β-dystrogycan proteolysis occurs in response to synaptic activity (Michaluk et al., 2007). MMP-9 catalyzes the cleavage within the β-dystroglycan ectodomain producing a 30 kDa C-terminal fragment and an N-terminal region that is further processed (Bozzi et al., 2015). The consequences of β-dystroglycan cleavage are not known, however, a growing body of evidence suggests a functional role for the entire dystrophin-glycoprotein complex at central synapses and in their plasticity since deletion of this protein is critically associated with attenuated high frequency stimulation-induced long-term potentiation at certain synapses (Satz et al., 2010).

There are several classes of extracellularly acting proteases that are modulated by synaptic activity and are well established to be engaged in synaptic plasticity (Qian et al., 1993; Lüthi et al., 1997; Lukasiuk et al., 2011; Huntley, 2012). The most extensively studied in the context of trans-synaptic CAMs cleavage are the families of matrix metalloproteinases (MMPs) and ADAM metalloproteinases, both belonging to the class of zinc-dependent proteases (metzincins) (Mott and Werb, 2004; Rivera et al., 2010; Huntley, 2012). Among MMPs, MMP-9 has been particularly intensively investigated in synapse function (Huntley, 2012; Vafadari et al., 2016). One of the first associations between MMP-9 and trans-synaptic machinery emerged upon experimental identification of β-dystroglycan (postsynaptic CAM identified as neurexin partner in the brain) (Sugita et al., 2001; Michaluk et al., 2007; **Figure 1**).

Apart from metaloproteinases, trypsin-type serine proteases are also well-evidenced to be involved in synaptic plasticity, with a particular involvement of neuropsin (KLK8) in modulation of trans-synaptic complexes (Baranes et al., 1998; Tamura et al., 2006).

One can easily notice that any alterations in this complex and proteolytically modulated trans-synaptic engineering might result in serious neuronal dysfunctions in developing or mature synapses. Indeed, scientific investigations on neuronal cultures and *in vivo* animal models have identified several potentially important trans-synaptic CAMs and their associated proteases and linked them to synaptic dysfunctions. Due to the large number of molecules and considering the fact that experimental evidence supporting the involvement of some direct trans-synaptic interactions and their digestion in synapse function is sometimes compelling, we wish to acknowledge here only the most exemplary ones (summarized in **Table 1**).

**Table 1:** Trans-synaptic molecules, their partner proteins, and associated proteases involved in synaptic function.

Family	Molecule	Transsynaptic partner and type of iteraction	Reference	Complex-associated extracellural proteases	Reference
Neuroligin	Neuroligin-1 neuroligin-2 neuroligin-3 neuroligin-4	β-Neurexin (direct interaction)	Ichtchenko et al., 1995, 1996; Gangwar et al., 2017	MMP-9 ADAM10	Peixoto et al., 2012; Suzuki et al., 2012
Cadherin	N-cadherin	N-cadherin (direct interaction) neuroligin-1 (through a S-SCAM protein)	Stan et al., 2010; Aiga et al., 2011	ADAM10 MT5-MMP calpain	Covault et al., 1991; Monea et al., 2006; Jang et al., 2009; Malinverno et al., 2010
Eph receptor	EphA1-10 receptors EphB1-5 receptors	EphrinA (direct interaction) ephrinB (direct interaction)	Klein, 2009; Daar, 2012	ADAM10 MMP-2 MMP-9 neuropsin	Hattori et al., 2000; Klein, 2004; Murai and Pasquale, 2004; Lin et al., 2008; Attwood et al., 2011
NCAM	NCAM	NCAM (direct interaction)	Bukalo and Dityatev, 2012	ADAM17 MMP-2 MMP-9 calpain	Covault et al., 1991; Kalus et al., 2006; Shichi et al., 2011
Contactins	Contactin-1 Contactin-2 Contactin-3 Contactin-4 Contactin-5 Contactin-6	Contactin-1 (direct interaction) Contactin-2 (direct interaction) Contactin-3 (direct interaction) Contactin-4 (direct interaction) Contactin-5 (direct interaction) Contactin-6 (direct interaction)	Zuko et al., 2013	_	_
Contactins	Contactm-3 Contactm-4 Contactin-5 Contactin-6	PTPRG (not studied)	Nikolaienko et al., 2016	_	_
LRR proteins	NGL-1 NGL-2	Netrin-G1 (direct interaction) Netrin-G2 (direct interaction)	Lin et al., 2003; Kim et al., 2006	_	_
LRR proteins	SALMs	SALMs (direct interaction)	Seabold et al., 2008	_	_
LRR proteins	LRRTMs	α-neurexin (not studied) β-neurexin (not studied)	de Wit et al., 2009; Ko et al., 2009; Siddiqui et al., 2010; Südhof, 2017	_	_
Dystroglycan	β-Dystroglycan	β-neurexin (through an α-dystrogjycan)	Sugita et al., 2001; Michaluk et al., 2007	MMP-9	Michaluk et al., 2007
Neurexophillins	Neurexophillin-1 Neurexophillin-2 Neurexophillin-3 Neurexophillin-4	α-neurexm (not studied)	Südhof, 2017; Chamma and Thoumine, 2018	_	_
Cerebellins	Cerebellin-1 Cerebellin-2 Cerebellin-3 Cerebellin-4	α-neurexin (not studied) β-neurexin (not studied)	Südhof, 2017; Chamma and Thoumine, 2018	_	_
Calsyntenins	Calsyntenins-3	α-neurexm (not studied)	Südhof, 2017; Chamma and Thoumine, 2018	_	_

### Neuroligins and Neurexins

**FIGURE 2:**
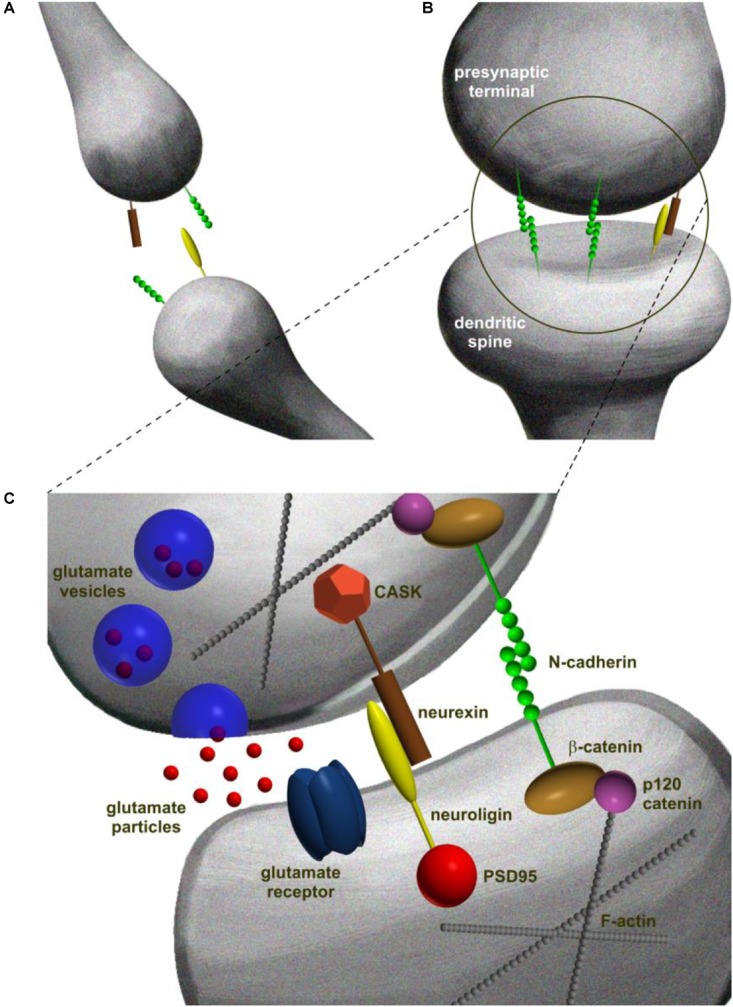
Schematic representation of different steps in trans-synaptic complex formation in excitatory synapse development. **(A)** Synaptogenesis is initiated at the points of contacts between dendritic filopodia and axons. At this stage, synaptic CAMs are distributed evenly at the contact point. **(B)** Contact point is stabilized by dimerization of the opposing CAM molecules that form trans-synaptic complexes spanning the partner cells and creating mechanical adhesion points. **(C)** Adhesion points develop into mature synapses that consist of presynaptic terminal with neurotransmitter vesicles and a postsynaptic density characterized by the presence of receptors. The molecular diversity of pre- and postsynaptic constituents is built upon C-terminal interactions of trans-synaptic complexes linking the trans-synaptic dimer to the scaffolding proteins and filamentous actin (McMahon and Díaz, 2011).

Trans-synaptic adhesion system is based on either a homo- or a heterophilic interactions between CAMs over the width of synaptic cleft (Yamada and Nelson, 2007). A canonical example of heterophilic interaction is the neuroligin/neurexin complex (Ichtchenko et al., 1995). Neurexins are presynaptic cell surface proteins that are expressed in neurons in plethora of alternatively spliced and regulated isoforms (Krueger et al., 2012). In vertebrates, neurexins genes possess two promoters that independently guide synthesis of α-neurexins and β-neurexins (Missler and Südhof, 1998). For ligand binding, the extracellular tail of α-neurexins contains six LNS (laminin-neurexin-sex hormone binding globulin) domains, as well as three EGF (epidermal growth factor-like) domains. On the other hand, the ectodomain of β-neurexins is composed of unique LNS motif (Reissner et al., 2013). Neuroligins are a family of four type-1 membrane postsynaptic CAMs (named and numbered as neuroligin-1 to neuroligin-4) that contribute to synapse specification. Neuroligins are composed of several domains, including extracellular region, a single transmembrane domain, and cytoplasmic tail. The extracellular region of neuroligins contains a cholinesterase-like domain, responsible for binding to certain splice variants of neurexins through their LNS domain (Lisé and El-Husseini, 2006). The cytoplasmic domains of neuroligins and neurexins contain a four amino acid-long C-terminal motif for binding PDZ domains of scaffolding proteins. Via this motif, neuroligins bind to postsynaptic intracellular scaffolding proteins, including PSD95 and many others (Ichtchenko et al., 1996; Irie et al., 1997; Meyer et al., 2004; Gangwar et al., 2017). The same time the presynaptic differentiation is coordinated through the PDZ-domain-binding motif of β-neurexin which interacts with the CASK (Hata et al., 1996) and Mint (Biederer and Südhof, 2000) proteins at the presynaptic site -both associated with Munc18 protein that is substantial for release of neurotransmitter (Verhage et al., 2000; **Figure 2**).

As demonstrated by elegant studies on heterogenic cultures, the aforementioned interactions seem to be sufficient for the synaptic arrangement. For instance expression of neuroligins in non-neuronal cells evoked differentiation of presynaptic constituent at the axonal processes of co-cultured neurons. On the other hand, expression of β-neurexin in non-neuronal cells triggered development of postsynaptic specification at the dendrites of neighboring neurons (Gokce and Südhof, 2013). Moreover, elevated expression of neuroligins in neuronal culture increased the number of synapses in respect of synapse type and neuroligin isoform (Chih et al., 2004, 2005; Varoqueaux et al., 2004; Levinson and El-Husseini, 2005). Furthermore, *in vivo* studies on knockout (KO) animals demonstrated that neuroligins as well as neurexins are crucial for the formation of synaptic contacts and their function (Missler et al., 2003; Varoqueaux et al., 2006). Later on, it was clarified that neuroligins have a significant role in balancing the number of excitatory and inhibitory neuronal contacts. To be precise, Song et al. (1999) showed by *in vitro* experiments that neuroligin-1 is specific for excitatory while Varoqueaux et al. (2004) demonstrated that neuroligin-2 is specific for inhibitory synapses. On the other hand, neuroligin-3 and -4 seem to be present on both types of synaptic junctions (Budreck and Scheiffele, 2007; Hammer et al., 2015).

Multiple pieces of data on neuroligin/neurexin complex role at synapses come from loss of function studies. For instance, it was shown that knocking out neuroligin-1 in mice attenuates the strength of excitatory synapses, whereas neurons lacking neuroligin-2 are characterized by attenuated efficacy of inhibitory connections. Moreover, the triple KO of neuroligin-1–3 in mice occurred to be neonatally lethal, and considerable synaptic deficits had been reported based on both *in vitro* and *in situ* studies of these animals (Varoqueaux et al., 2006). Moreover, silencing neuroligin-1 in mice blocks the storage of fear memory by reducing NMDAR-mediated currents and affects the expression of long-term potentiation (LTP) (Kim et al., 2008). In addition, NMDAR/AMPAR current ratio and LTP were found reduced in neuroligin-1 KO mice (Chubykin et al., 2007). On the other hand, overexpressing neuroligin-1 also leads to the learning and memory deficits and impaired synaptic plasticity (Dahlhaus et al., 2009). Neuroligin-2 overexpressing mice show increased synaptic contact area and enlarged pool of reserve vesicles in synapses, and alterations in the excitation to inhibition ratio which seems to result in anxiety and loss of social interactions (Hines et al., 2008). In addition, neurexins in conjunction with neuroligins also contribute to the synaptic plasticity throughout the activity-dependent molecular pathways (Varoqueaux et al., 2004; Jedlicka et al., 2015). Experimental data show that the extracellular domain of neuroligin-1 can be cleaved by MMP-9 and ADAM10, and the remaining membrane-bound peptide is subsequently processed by γ-secretase. The shedding of neuroligin-1 requires NMDA receptor activity (Peixoto et al., 2012; Suzuki et al., 2012).

Studies have demonstrated that besides neuroligins, neurexins create trans-synaptic interactions also with several other proteins, particularly, neurexophilins, cerebellins, calsyntenins, as well as dystroglycans, and LRRTMs (Südhof, 2017; Chamma and Thoumine, 2018). Briefly, neurexophilins comprise a family of four secreted glycoproteins (neurexophilin-1 to neurexophilin-4) (Missler et al., 1998) that play an essential function in synaptic plasticity (Born et al., 2014; Neupert et al., 2015). They were initially discovered as an α-neurexin partners by affinity chromatography (Petrenko et al., 1996). Cerebellins are a group of secreted glycoproteins that were classified to the C1q and TNF family (Kishore et al., 2004). There are four vertebrate cerebellins family members (named cerebellin-1 to cerebellin-4) that are abundantly expressed in the brain. They critically contribute to the synaptic function and plasticity through the trans-synaptic interactions with presynaptic neurexins and postsynaptic glutamate receptors (Ito-Ishida et al., 2008; Uemura et al., 2010; Matsuda and Yuzaki, 2011; Seigneur and Südhof, 2017). Their important role in synaptic organization has been very recently confirmed with studies on a single, double, and triple constitutive cerrebellin KO mice (Seigneur and Südhof, 2018). Calsyntenins are members of the cadherin superfamily, type I transmembrane proteins containing two cadherin repeats, laminin, neurexin, and LNS domains (Vogt et al., 2001; Araki et al., 2003). Among them, novel synaptic organizer named calsyntenin-3 has been recently reported by Pettem et al. (2013). Calsystenin-3 localizes predominantly postsynaptically (Hintsch et al., 2002) and was characterized by its ability to bind to α- but not β-neurexins (Lu et al., 2014). The protein seems to organize both excitatory and inhibitory synaptic assembly as demonstrated by co-culture assay (Pettem et al., 2013). This action was suppressed once calsyntenin-3 extracellular domain was cleaved (Pettem et al., 2013). Due to broadly documented contribution to trans-synaptic interactions dystroglycans and LRRTMs were described in a separate sections of this review.

### Cadherins

A common example of prototypic homophilic interactions is cadherin/cadherin complex. Cadherins are large family of glycosylated transmembrane cell–cell adhesion molecules conserved among multicellular organisms, which play essential roles in tissue morphogenesis (Hulpiau and van Roy, 2011).

N-cadherin (neural cadherin) is one of the most abundant cadherins in synapses. It belongs to the classic cadherins – a single-pass transmembrane proteins containing five extracellular cadherin domains, a transmembrane motif, and a cytoplasmic domain. The protein homophilic adhesion is calcium-dependent (Takeichi, 2007). Molecular determinants of N-cadherin association with the synapse are based on extracellular domain of the protein which binds to the other N-cadherin molecule on juxtaposed cell membrane and highly conserved C-terminus binding β-catenin and p120-catenin (Rubio et al., 2005; Elia et al., 2006; Takeichi, 2007). β-catenin recruits α-catenin (a binding partner of filamentous actin) to the complex and plethora of PDZ domain-containing proteins (Arikkath and Reichardt, 2008). This mesh of interactions is considered to guide structural and functional organization of synaptic assembly linking N- cadherin to cytoskeletal elements (Yamada et al., 2005), ensuring the adhesion of synaptic membranes, organizing the presynaptic vesicle clusters (Bamji et al., 2003; Bozdagi et al., 2004), and postsynaptic membrane machinery (Togashi et al., 2002; Tai et al., 2008) together with the glutamate receptors at the synapse (Schnell et al., 2002; Danielson et al., 2012; **Figure 2**). The above notions have been supported by many studies on neuronal cultures. For instance, cells lacking either N-cadherin or β-catenin showed multiple synaptic malformations such as more filopodia-like thinner spines with parallel decrease of total spine number (Okuda et al., 2007; Saglietti et al., 2007; Mendez et al., 2010).

Alteration of N-cadherin function induces detectable synaptic deficits that clearly demonstrate structural and functional association of N-cadherin with the processes of synapse stabilization, synaptic transmission and plasticity at both sides of the synapses. For example conditional KO mice are characterized by reduced LTP and impaired spine enlargement as demonstrated by Bozdagi et al. (2010) in the CA1 hippocampal region. The LTP-induced long-term stabilization of synapses was reported to be impaired when N-cadherin is knocked down (Mendez et al., 2010). The lack of N-cadherin markedly diminished short-term facilitation and depression at excitatory synapses (Jungling et al., 2006). Moreover, presynaptic N-cadherin adhesion, when reduced, leads to the altered recycling of vesicles (Togashi et al., 2002). On the other hand, overexpression of the N-terminal domain of N-cadherin seems to be responsible for the increased frequency of miniature excitatory postsynaptic currents (mEPSCs) (Saglietti et al., 2007). Interestingly, cooperation between neuroligin-1 and N-cadherin had also been inspected and reported to be important for the formation of excitatory synapses and control of vesicle dynamics at synapses (Stan et al., 2010; Aiga et al., 2011). N-cadherin extracellular domain can be processed by ADAM10 (Malinverno et al., 2010) or membrane type 5 MMP (MT5-MMP) in an AMPA-dependent manner (Monea et al., 2006). The continuation of that molecular events is the cleavage of N-cadherin intracellular fragment which is processed by γ-secretase in order to release a C-terminal-derived motif that affects CREB dependent transcription (Marambaud et al., 2003).

### Eph Receptors and Ephrins

Eph receptors are family of receptor tyrosine kinases, containing an extracellular region with the ephrin ligand-binding domain, a single transmembrane segment, and a cytoplasmic domain containing PDZ-binding motif (Pasquale, 2004, 2005). The family is comprised of 10 EphA and 5 EphB receptors which have been studied in various physiological processes (Kullander and Klein, 2002; Himanen, 2012). EphA and EphB receptors are characterized by the affinity to transmembrane glycosylphosphatidylinositol-anchored proteins ephrinA, and ephrinB, respectively. Eph receptors and ephrins were found at both pre- and postsynaptic sites (Klein, 2009). Resultant Eph receptor/ephrin interaction mediates adhesion and bidirectional communication between the receptor-expressing membrane and the ephrin-expressing partner membrane (Daar, 2012). EphrinA-mediated EphA signaling modulates the synaptic plasticity (Hruska and Dalva, 2012). In particular, the activation of EphA4 downregulates the synaptic GluR1, and attenuates amplitude of mEPSC (Fu et al., 2011). Moreover, the EphA4-deficiency in hippocampal CA1 region was found by Grunwald et al. (2004) to alter maturation of dendritic spines (Murai et al., 2003), to impair LTP as well as long-term depression (LTD). Also EphrinB-mediated EphB signaling seems to be important for synaptic functions. In particular, the EphB1-3 receptors expression reduces the number of glutamergic synapses and the recruitment of NMDARs and AMPARs (Henkemeyer et al., 2003). Also in another study localization of the AMPARs was shown to be modulated by EphB2 (Kayser et al., 2006). As demonstrated in cultured neurons, siRNA-mediated downregulation of EphB2 expression in the postsynaptic neuron reduced mEPSCs frequency at postsynaptic cell (Kayser et al., 2006). In addition, mice deficient in EphB2 are characterized by reduced NMDAR-mediated currents and impaired LTP (Henderson et al., 2001). Later on, it occurred that EphrinB2 is essential for maintaining both LTP and LTD through the C-terminal PDZ interaction (Bouzioukh et al., 2007). In CA1 neurons of ephrinB3-deficient mice, mEPSCs show reduced amplitude. The same time NMDAR/AMPAR ratio is increased in this animal (Antion et al., 2010). In CA3 pyramidal cells extracellular application of soluble ephrinsB reduces mossy fiber LTP (Contractor et al., 2002). Replacement of ephrinB3 intracellular domain with β-galactosidase blocks the LTP at mossy fibers (Armstrong et al., 2006). The Eph receptors/ephrins interactions can be decomposed as a result of proteolytic cleavage of ephrinA2 and ephrinB2 by ADAM10 (Hattori et al., 2000). Moreover, it has been demonstrated *in vitro* that EphB2 receptor can also be cleaved by MMP-2 and MMP-9 in dissociated hippocampal neuronal cultures (Lin et al., 2008). Finally, the Ephrin/Eph receptor complex was demonstrated to be involved in cognition when cleaved by MMP-9 following induction of hippocampal LTP (Klein, 2004; Murai and Pasquale, 2004). Another study reported neuropsin-mediated cleavage of EphB2 in amygdala which was related to stress-induced synaptic plasticity and anxiety (Attwood et al., 2011).

### NCAM

The neural cell adhesion molecule (NCAM) is a glycoprotein which expression was reported at both pre- and postsynaptic constituents. The N-terminal region of NCAM binds to the partner NCAM molecule and contains five Ig domains followed by a region containing two fibronectin type III-like repeats (Kasper et al., 2000). Numerous alternatively spliced variants of NCAM were identified in rat brain (Reyes et al., 1991) and studies have demonstrated that NCAM has an influence on synapse structural and functional maturation through both homo- and heterophilic interactions (Bukalo and Dityatev, 2012). For instance, postsynaptic NCAM forms a complex that is responsible for the recruitment of NMDARs to synapses, and is crucial for NMDAR-dependent forms of plasticity such as LTP and LTD (Müller et al., 1996; Bukalo et al., 2004; Sytnyk et al., 2006). NCAM might be polysialylated. Polysialylation is a posttranslational modification of the NCAM. In the adult brain, polysialylated NCAM (PSA-NCAM) is restricted to regions of neuroplasticity where it affects homophilic NCAM binding and modulates NCAM-mediated adhesion (Rutishauser and Landmesser, 1996; Kiss and Rougon, 1997). The ablation of NCAM results in reduction of the number of synapses (Dityatev et al., 2000). Moreover, suppression of NCAM-mediated adhesion with either synthetic peptides or function-interfering antibodies reduced LTP in CA1 region, along with impaired hippocampal-dependent learning. Hinkle et al. (2006) have revealed that NCAM might be processed by ADAM17, MMP-2, and MMP-9.

### Contactins

Contactins are Ig-CAMs that contain six N-terminal Ig-like domains (Buttiglione et al., 1996; Perrin et al., 2001). There are six members of this subfamily that are typically expressed in the nervous system: contactin-1 (contactin/F3), contactin-2 (also named TAG-1), contactin-3, and contactin-4 (BIG-1 and BIG-2, respectively), and contactin-5, contactin-6 (NB-2 and NB-3) (Shimoda and Watanabe, 2009). Contactins function most probably at pre- and postsynaptic sites through predominantly heterophilic interactions with distinct members of contactin family and other trans-synaptic CAMs such as protein-tyrosine phosphatase receptor type G (PTPRG) (Zuko et al., 2013; Nikolaienko et al., 2016), however, complete recognition of their trans-synaptic activities requires detailed studies. Among contactins, contactin-1 has been extensively studied in context of synaptic function. Contactin-1 has been identified at postsynaptic density of CA1 pyramidal cells and its inactivation impairs paired-pulse facilitation and LTD. Synapses deficient in contactin-1 show decreased EPSCs amplitudes upon low-frequency stimulation (Murai et al., 2002). Contactin-4 and 5 were found to be involved at early stages of development in synapse differentiation (Mercati et al., 2013) whereas contactin-6 deficient mice show reduced number of excitatory synapses but not inhibitory ones (Sakurai et al., 2010). Very few if any studies directly address the prospective proteolytic cleavage of contactins at the synapse. Thus further investigation is required to find out their enzymatic partners.

### LRR Proteins

Several types of LRR (leucine-rich repeats) proteins were identified at synapses, in particular NGLs (netrin-G ligands), SALMs (synaptic adhesion-like molecules), and LRRTM (leucine rich repeat transmembrane) proteins. The three NGLs expressed in vertebrates (NGL1-3) are type I membrane proteins localized to postsynaptic sites. Their name comes from the NGLs characteristic potential to bind to presynaptic netrins (netrin-G1 and -G2) (Lin et al., 2003; Kim et al., 2006). Crucial for this heterophilic interactions are NGLs extracellular leucine-rich repeats, and their single extracellular Ig-domain. At the intracellular side a short cytoplasmic tail of NGLs is capable of interacting with PSD-95 (Woo et al., 2009). On the other hand netrin-G1 and -G2 isoforms are associated with axonal terminals by a GPI anchor (Nakashiba et al., 2002). Mice lacking netrin-G/NGL trans-synaptic system components develop behavioral deficits (Zhang et al., 2008).

Synapse-organizing functions were also reported for SALMs (Nam et al., 2011). Like NGLs, SALMs are type I membrane proteins. They contain crucial amino-terminal leucine-rich repeats, and a single Ig-domain. The complete role of SALMs in synapses remains unclear, however, it has been reported that at the extracellular side a subset of SALMs form homophilic complexes (Seabold et al., 2008), and they may bind AMPAR and NMDAR receptors or PSD-95 (Ko et al., 2006; Wang et al., 2006).

LRRTMs have also been reported to play a synaptic function. A plausible role of LRRTM proteins in synaptogenesis was first identified bioinformatically (Laurén et al., 2003), and then confirmed experimentally during synapse formation (Linhoff et al., 2009). The mechanisms of LRRTM-based trans-synaptic interactions remain underinvestigated. Studies revealed that LRRTM proteins bind to neurexins in a selective manner (de Wit et al., 2009; Ko et al., 2009; Siddiqui et al., 2010). Deficits of LRRTM proteins do not seem to exert any considerable phenotypes (Linhoff et al., 2009). Nevertheless knockdown of LRRTMs expression *in vitro* and *in vivo* is associated with decreased AMPAR trafficking (Ko et al., 2011; Soler-Llavina et al., 2011). There is limited experimental data on LLR protein cleavage at the synapse. Therefore that phenomenon must be further addressed.

### Dystroglycan

Dystroglycan is an ubiquitous membrane protein and part of dystrophin-glycoprotein complex. It links the cytoskeleton to the extracellular matrix and stabilizes the dystrophin-glycoprotein complex at the membrane (**Figure 1**). Dystroglycan is a duplex of α- and β-subunits. The α-dystroglycan is a highly glycosylated extracellular component, whereas β-dystroglycan intermediates in interaction between α-subunit and the cytoskeleton. Dystroglycan, like neuroligin, also binds specifically to a subset of the LNS domains of neurexins (Sugita et al., 2001). The dystroglycan complex has been found to colocalize with GABAA receptors in the CNS and it was demonstrated that the surface expression of GABA receptors increases upon neuronal hyperactivity in a dystroglycan-dependent manner (Lévi et al., 2002; Pribiag et al., 2014).

## Trans-Synaptic Cams in Epileptogenesis and Epilepsy in Patients and Animal Models

The epilepsy comprises a very heterogeneous group of neuropsychiatric disorders. They are defined by the occurrence of recurrent seizure episodes (Fisher et al., 2014). In addition to seizures, epilepsy is usually associated with cognitive impairments (Berg and Scheffer, 2011). Epilepsy is also common comorbidity of various mental conditions, such as autism spectrum disorders or schizophrenia. The classification of epilepsies has been recently revised by the International League against Epilepsy (Berg et al., 2010; Fisher et al., 2017).

Comprehensive understanding of these mechanisms cannot be fully acquired without use of animal models. The animal models of epilepsy provide a clinically relevant context for exploring the seizure-related structural and functional changes of neuronal networks during the course of epileptogenesis. Different animal models represent different forms of human epilepsies. Extensively used are models mimicking temporal lobe epilepsy (TLE) the most common form of epilepsy in adults. Rodents with recurrent TLE seizures are typically generated with use of chemoconvulsants such as pilocarpine, or kainate (Leite et al., 2002). Animals treated with the chemoconvulsive drugs display initial seizure insult (*status epilepticus*) that leads to a latent period and eventually to the occurrence of chronic manifestation of unprovoked tonic-clonic seizures associated with histopathological changes that are characteristic for TLE (Lothman and Bertram, 1993; Mathern et al., 2002). Like *status epilepticus*-induced TLE, electric kindling may also reproduce chronic epileptogenic features in temporal lobe. Electric kindling is an epileptiform plasticity phenomenon in which repetitive administration of sub-convulsant electrical stimuli in a specific brain region can lead to progressive development of seizures, culminating in generalized seizure activity (McNamara, 1984). Kindling is also often induced chemically by the repeated administration of subconvulsive dose of pentylenetetrazol (PTZ).

Post-traumatic epilepsy (PTE) is one of the leading causes for acquired epilepsies in adults. PTE develops often as a result of traumatic brain injury (TBI) caused by external mechanical force. This initial brain insult triggers cascade of molecular changes that leads to the appearance of spontaneous recurrent seizures (Pitkänen and Lukasiuk, 2011). Considering the high heterogeneity of TBI in humans, TBI animal models are very diverse in order to recapitulate human syndrome as completely as possible (Pitkänen et al., 2009).

Over the last years, many genes with effect on human epilepsy have been reported providing important evidence for heritability of the condition (Ottman et al., 2010). There are several genetic animal models that resemble idiopathic epilepsy in humans. Genetically established mice and rat strains have been created by selective breeding of seizure-susceptible individuals (Frankel, 2009). As a result in these genetic animal models, seizures are either induced by specific sensory stimulation or occur spontaneously (Serikawa et al., 2015).

Distinct origins of the condition and associated seizure types are reflected by the very diverse and complex molecular mechanisms underlying epilepsies. Given the accumulating research evidence based on animal models epilepsy is now clearly recognized also as a synaptopathy. Synaptic alterations contribute to the epilepsy development, i.e., epileptogenesis, recurrent seizure initiation and propagation, and associated cognitive deficits. This notion is further supported by clinical studies suggesting that several of these synaptic mechanisms might involve dysfunctions in trans-synaptic adhesion.

### Neuroligins and Neurexins

Since neuroligin/neurexin interaction regulates the proper balance between the number of excitatory and inhibitory synapses, it is not surprising that their alterations are important epileptic hallmarks. For instance, it was shown that neuroligin-1 and its binding partner neurexin-1β are intensively expressed in epileptic foci of human patients with TLE (Fang et al., 2016). Further, the animal studies revealed that neuroligin-1 knockdown prolonged chronic seizure onset and reduced seizure severity in epileptic rats. In addition patch-clamp recordings of whole-cell in brain slices from neuroligin-1 knockdown epileptic rats revealed a decrease frequency of action potentials and miniature postsynaptic currents generation in excitatory cells of hippocampal network. In this neuron the amplitude of NMDAR-dependent excitatory postsynaptic currents were also attenuated and general NMDAR1 subunit expression was found reduced in the hippocampus of epileptic rats (Fang et al., 2016). Moreover genomic microarray test detected the neuroligin-1 deletion in a patient with seizure disorder (Millson et al., 2012) and mutations in neuroligin-4 have been also associated with convulsions and synaptic dysfunctions (Laumonnier et al., 2004). Mutations in neurexin-1, a neuroligin partner were also found in patients with epilepsy. Neurexin-1 deletions result in an early onset epilepsy associated with severe recessive mental retardation (Harrison et al., 2011). Furthermore, a genome-wide study on patients with idiopathic generalized epilepsy had revealed that the risk of syndrome is increased upon exon-disrupting deletions of neurexin-1 (Møller et al., 2013). Studies on animals revealed that the level of neurexin-2 expression was enhanced in the dentate gyrus upon seizures (Górecki et al., 1999).

### Cadherins

The involvement of classical cadherins in epileptogenesis and epilepsy has not apparently been examined experimentally. Nevertheless, studies on animal models revealed a potentially pathogenic mechanism related to N-cadherin. N-cadherin expression was reported to be increased in hippocampus up to 1 month following pilocarpine-induced seizure and was found consistent with the induction of mossy fiber sprouting (Shan et al., 2002).

Most of the epilepsy incidents are idiopathic or acquired, however, there is a strong evidence for heritability of the condition. Important non-classic cadherin implicated in genetic cases of epilepsy is protocadherin-19 (PCDH19). Protocadherins possess strong structural similarity to classic cadherins particularly, they contain extracellular motifs that are homologous to those of classic cadherins. The name protocadherin suggest an evolutionary old group of molecules, however, protocadherins have very late origin and for the first time appear in primitive vertebrates (Weiner and Jontes, 2013). The PCDH19 mutations are responsible for female-restricted epilepsy with mental retardation (EFMR) (for a review, see Depienne and LeGuern, 2012) that has been classified as infantile familial or sporadic epilepsy in female patients with or without intellectual disability (Dibbens et al., 2008). Due to its female-limited expression PCDH19 gene escaped epilepsy mapping until systematic sequencing of X-chromosome exons clearly revealed mutations of the PCDH19 gene as the cause of the disorder. PCDH19 alterations affect heterozygous females while hemizygous males are spared (Dibbens et al., 2008). For example, in this context genomic deletions of PCDH19 have been clinically identified in two unrelated girls with seizures (Vincent et al., 2012). Most typically PCDH19-related epilepsy is characterized by early-onset of seizures (6–36 months) followed by recurrent seizures appearing throughout childhood (Scheffer et al., 2008).

The strong association of PCDH19 with epilepsy inheritance is also stressed by genetic screening demonstrating that 11 out of 45 patients suffering from Dravet syndrome (severe myoclonic epilepsy of infancy) carried mutations in PCDH19 (Depienne et al., 2009). Moreover, mutations in PCDH19 have also been identified in many female patients with febrile seizures and in a wide spectrum of other cases (Jamal et al., 2010; Marini et al., 2010; Specchio et al., 2011a,b; Higurashi et al., 2012).

#### Eph Receptors and Ephrins

The expression pattern and cellular distribution of EphB receptor/ephrinB complex was investigated in patients with intractable TLE and correlated with data obtained from lithium-pilocarpine-treated rats (Huang et al., 2016). In comparison to control groups, ephrinB3 and EphB3 mRNA expressions were significantly up-regulated in patients and rats with chronic TLE seizure. Western blot analysis and semi-quantitative immunohistochemistry showed that ephrinB3 and EphB3 protein level were up-regulated in both patients and affected animals. Similarly, increased expression of EphA10 receptor as well as ephrinA4 was reported upon pilocarpine induced acute seizures, suggesting EphA10, ephrinA4 involvement in epileptogenesis (Xia et al., 2013). In addition to that, EphA5 and ephrin-A3 were reported to delay development of chronic seizures induced with stimulation of performant path fibers (Xu et al., 2003).

### NCAM

NCAM-1 concentration in cerebrospinal fluid was found elevated in epilepsy patients in comparison to healthy individuals. Intensive NCAM-1 content was also detected in drug-resistant epilepsy patients when compared to the drug-effective patients (Wang et al., 2012). Therefore, NCAM-1 concentration in cerebrospinal fluid could be considered a biomarker for epilepsy. NCAM association with epilepsy was also confirmed in animal studies. Particularly, it was revealed that NCAM-positive neurons were observed in hippocampus of rats that had experienced repeated kindled generalized seizures (Sato et al., 2003).

To explore the role of polysialylated-NCAM in epilepsy, endoneuraminidase-based inactivation of PSA-NCAM was performed by administration of the drug into the contralateral ventricle of mice with chronic seizure. That action induced neurodegeneration of cells in dentate gyrus and CA3 region of hippocampus and promoted the early onset of focal seizures (Duveau and Fritschy, 2010). Another studies focused on the impact of PSA-NCAM on pathophysiology of epilepsy demonstrated that loss of PSA-NCAM decreased the number of hippocampal newborn cells in kindling-associated changes of hippocampal neuronal network. The downregulation of the hippocampal cell proliferation rate as a consequence of PSA removal did not affect the kindling progression which occurred to be comparable in rats with and without removal of PSA. On the other hand, loss of PSA increased acute seizure susceptibility as indicated by reduced seizure thresholds before kindling (Pekcec et al., 2007). Continuation of the studies on the role of PSA-NCAM utilized an electrically induced poststatus epilepticus (SE) rat model. That approach reviled that loss of PSA counterbalanced the SE-induced increase in neurogenesis in a significant manner. In addition, transient lack of PSA during induction of SE or during the initial period of epileptogenesis exhibited a gentle cognition alteration as revealed by the trials in Morris water maze (Pekcec et al., 2008).

### Contactins

Contactin-6 deletions in humans were reported to be associated with various neurological syndromes including seizures as a comorbidity (Hu et al., 2015; Juan-Perez et al., 2018). This strongly suggests implication of contactin-6 in epilepsy, however, further studies on animal models are essential to test this hypothesis.

### LRR Proteins

SALM3 has been lately implicated in epileptogenesis and epilepsy. The study by Li et al. (2017) showed that SALM3 was significantly overexpressed in epileptic rats in two distinct animal models (lithium-pilocarpine model, and PTZ kindling model) when compared to control animals. Further Inhibition of SALM3 by SALM3 shRNA ameliorated *status epilepticus* in the acute phase and decreased spontaneous recurrent seizures evoked by pilocarpine. Inhibition of SALM3 also prolonged the latent period in the PTZ kindling model. The aforementioned results suggest that SALM3 plays a role in epileptogenesis and that its suppression might exert therapeutic effect.

### Dystroglycan

Initially, Kaczmarek et al. (2002) reported that β-dystroglycan undergoes limited cleavage in response to kainate treatment in a model of rat temporal lobe epileptogenesis. This finding was further elaborated by Michaluk et al. (2007), who demonstrated that seizure-evoked limited proteolytic cleavage of β-dystroglycan was MMP-9 dependent. Furthermore, Gondo et al. (2014) demonstrated *in vitro* that abnormal epileptiform activation of neurons with prolonged zero magnesium conditions decreased the expression of β-dystroglycan in mice cortical brain slices. The authors also showed that the β-dystroglycan deficit was associated with the dysfunctions of astrocytic endfeet which suggests epileptogenic downregulation of β-dystroglycan at blood-brain interface. Interestingly, clinical studies have identified two girls with alpha-dystroglycanopathy who presented severe and farmaco-resistant epilepsy characterized with electrographic myoclonic seizures (Di Rosa et al., 2011).

### Trans-Synaptic CAMs-Related Proteases in Epilepsy

Among extracellular proteases the most intensively studied in epilepsy pathogenesis are metzincin metalloproteinases. Some of them have already had an identified trans-synaptic target which makes metzincins potential contributors to aberrant synaptic plasticity that occurs in epilepsy development.

The best examined metzincin that is associated with trans-synaptic CAM and plays the significant role in different models and types of epilepsy is MMP-9 (Bronisz and Kurkowska-Jastrzêbska, 2016; Vafadari et al., 2016). The very first reports concerning the participation of MMP-9 in epilepsy were provided by Zhang et al. (1998, 2000), who presented that MMP-9 levels increase in brain of the rats subjected to kainate treatment. Later on, Szklarczyk et al. (2002) showed that MMP-9 at the level of mRNA, protein and enzymatic activity were markedly increased by kainate in the hippocampus, the brain region spared from the cell loss and instead undergoing extensive aberrant plasticity, contributing to the epileptogenesis. In fact, this study founded the field of MMP-9 in the plasticity. Finally, Wilczynski et al. (2008) established the functional role of MMP-9 in epilepsy, demonstrating that: (i) MMP-9 protein levels and enzymatic activity were strongly increased at the synapse upon seizures; (ii) sensitivity to pentylenetetrazol-based kindling was decreased in MMP-9 KO mice, but was increased in transgenic rats with neuronal overexpression of MMP-9; (iii) MMP-9 deficiency diminished kainate-evoked epileptiform changes in reviring of hippocampal network.

Subsequently, the presumed role of MMP-9 in epileptogenesis was confirmed with use of other models namely treatment with either pilocarpine or 4-aminopyridine (Kim et al., 2009; Takács et al., 2010). Recently, Pijet et al. (2018) have demonstrated that MMP-9 deficiency decreased the seizure appearance whereas overexpression of MMP-9 increased the number of mice with spontaneous seizures in posttraumatic injury model. Studies on animals were supplemented with clinical data demonstrating that in human patients with different types of epilepsy, occurrence of chronic seizures is correlated with the elevated levels of MMP-9 in serum, plasma, and cerebrospinal fluid as well as in the brain (Leppert et al., 2000; Suenaga et al., 2008; Li et al., 2012; Romi et al., 2012; Konopka et al., 2013; Zybura-Broda et al., 2016). A role of MMP-9 in the development of epilepsy has been also supported by studies with genetic model based on Wistar Glaxo Rijswijk (WAG/Rij) rats (Kim et al., 2009; Takács et al., 2010).

The role of other MMPs in epilepsy development has been so far poorly investigated. Experimental data indicates the significant increase of MMP-2 activity in animal models of TLE after systemic injection of pilocarpine (Hoehna et al., 2012) or kainate (Zhang et al., 1998, 2000). Gorter et al. (2007) also demonstrated enhancement in the expression of MMP-2 and -14 induced by generalized seizure evoked by electrical stimulation of the hippocampus. In kainate-evoked chronic seizures, also MMP-3 expression was reported to be elevated (Penkowa et al., 2005; Lee et al., 2010). Interestingly, serum concentration of MMP-3 in epileptic patients was in contrast found to be decreased (Wang et al., 2016).

Apart from MMPs, other proteases targeting CAMs were also implicated in epileptogenesis and epilepsy. For instance, ADAM9 and ADAM10 expression were reported to be induced in dentate gyrus of hippocampus following kainite-induced status epilepticus (Ortiz et al., 2005). Moreover, mice with intensive ADAM10 overexpression experience more frequent seizures than either control animals, or mice with moderate ADAM10 overexpression (Clement et al., 2008). In addition, ADAM22- and ADAM23-deficient mice are characterized by occurrence of multiple spontaneous seizures (Sagane et al., 2005; Owuor et al., 2009; Fukata et al., 2010). Also ADAMTS1 expression was demonstrated to be increased after status epilepticus-evoked seizures (Gorter et al., 2007). Neuropsin (KLK8) downregulation has been reported to be associated with seizure burden in animal model of kainate-induced epilepsy (Missault et al., 2017).

There are also other types of proteases definitely implicated in cleavage of trans-synaptic CAMs, and with strong relation to epileptogenesis and epilepsy. Particularly worth to mention is for instance calpain -a protein that belongs to the family of calcium-dependent cysteine proteases (Saido et al., 1992) that was demonstrated to truncate N-cadherin and NCAM (Covault et al., 1991; Jang et al., 2009) and its functional role in epilepsy development was confirmed by a recent work of Lam et al. (2017). Nevertheless, these proteases are predominantly intracellular thus their detailed description is not in the scope of this review.

## Concluding Remarks and Perspectives

Although evidence implicating different trans-synaptic complexes in the formation and maintenance of synaptic structure is considerable and their role in synaptic dysfunction convincing, further experimental data is required in order to understand the biological consequences and significance of the proteolytic trans-synaptic CAMs cleavage in modulation of synaptic function. Initial studies have already started to shed light on the dynamics of the key trans-synaptic adhesion molecules cleavage products, in response to synaptic activity. Such experimental data will be essential for an in-depth understanding of the function of adhesion molecules in synaptopathies. Particularly in epilepsy where the trans-synaptic CAMs seem to be crucial for many epileptogenic mechanisms and therefore comprise promising targets for the future pharmacological therapeutic interventions.

## Author Contributions

All authors listed have made a substantial, direct and intellectual contribution to the work, and approved it for publication.

## Conflict of Interest Statement

The authors declare that the research was conducted in the absence of any commercial or financial relationships that could be construed as a potential conflict of interest.
